# Properties of Concrete Using Treated Low-Class Recycled Coarse Aggregate and Blast Furnace Slag Sand

**DOI:** 10.3390/ma13040843

**Published:** 2020-02-13

**Authors:** Yuji Miyazaki, Takeshi Watanabe, Yuji Yamada, Chikanori Hashimoto

**Affiliations:** 1 Civil Foundation division, Miyazaki Kiso Construction Co. Ltd., Tokushima 779-0222, Japan; 2 Concrete Engineering Laboratory, Department of Civil and Environmental Engineering, Tokushima University, Tokushima 770-8506, Japan; chika@ce.tokushima-u.ac.jp; 3 Construction Material Laboratory, Department of Civil Engineering, Fukuoka University, Fukuoka 814-0180, Japan; yyamada@fukuoka-u.ac.jp

**Keywords:** recycled coarse aggregate, blast furnace slag sand, resistance to freezing and thawing, bleeding capacity

## Abstract

Since high quality natural aggregates are becoming scarce, it is important that industrial recycled products and by-products are used as aggregates for concrete. In Japan, the use of recycled aggregate (RG) is encouraged. Since, strength and durability of recycled aggregate concrete is lower than that of normal aggregate concrete, the use of recycled aggregate has not been significant. In order to improve physical properties of concrete using recycled coarse aggregate, blast furnace slag sand has been proposed. Recently, blast furnace slag sand is expected to improve durability, freezing, and thawing damage of concrete in Japan. Properties of fresh and hardened concrete bleeding, compressive strength, and resistance to freezing and thawing which are caused by the rapid freezing and thawing test using liquid nitrogen is a high loader than the JIS A 1148 A method that were investigated. As a result, concrete using treated low-class recycled coarse aggregate and 50% or 30% replacement of crushed sand with blast furnace slag sand showed the best results, in terms of bleeding, resistance to freezing and thawing.

## 1. Introduction

In Japan, disposal sites have been decreasing due to active industrial works. In addition, resources of natural aggregate for producing concrete has been decreasing due to protection of the environment. Therefore, there the need for concrete which can reduce environmental impact by using aggregate from industrial by-products has been increasing. In particular, the use of recycled aggregate has been desired over recent years in Japan. In Japan, Industrial Standards (JIS), has three types of recycled aggregates which are classified as class H, class M, and class L. Legal improvement for practical use of recycled aggregate has been progressing steadily. However, concrete using recycled aggregate is rarely used on the market, because its standing in quality assurance and cost performance is hampered. Strength and durability of concrete using low quality recycled aggregates as JIS class M and L are also generally much lower than that of normal concrete due to high absorption and other factors [1,2,3,4,5].

Recent research in Japan, carried out by the Japan Society of Civil Engineers (JSCE) has been focusing on the use of blast furnace slag sand to produce more durable concrete. JSCE suggested that resistance to freezing and thawing is improved, drying shrinkage can be reduced, and resistance against chloride ion diffusion is improved [6]. In addition, recent research has examined its performance throughout the world [7,8,9,10,11,12,13,14]. Some research papers [15,16,17,18,19] have been published on concrete using recycled aggregate recently. However, there are few studies on the concrete using blast furnace slag sand and recycled aggregate used to make high-performance recycled aggregate concrete. In this study, in order to improve the strength and durability of concrete using low-class recycled coarse aggregate, blast furnace slag sand was employed. Several mix proportions were used and the concrete test pieces were examined for bleeding, compressive strength, and resistance to freezing and thawing.

## 2. Materials and Methods

### 2.1. Materials

**Figure 1:**
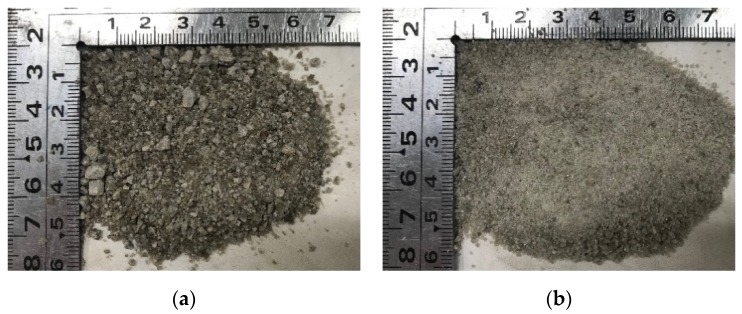
Blast furnace slag sand used: (**a**) BFS5 (5 mm in max. dia.); (**b**) BFS1.2 (1.2 mm in max dia.).

**Figure 2:**
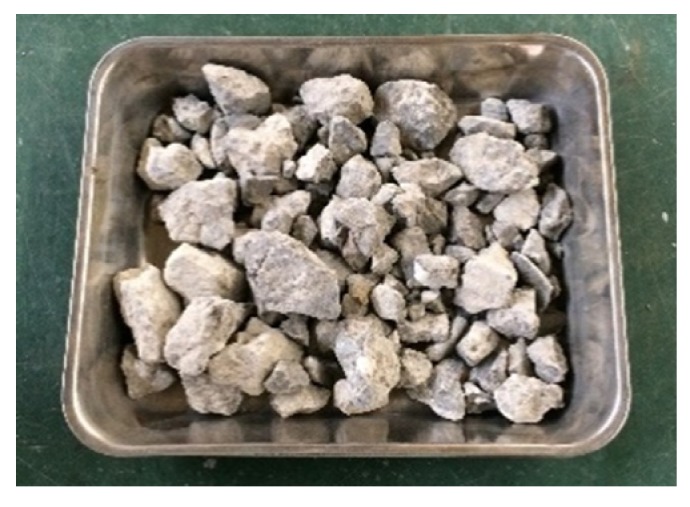
Recycled coarse aggregate used.

**Figure 3:**
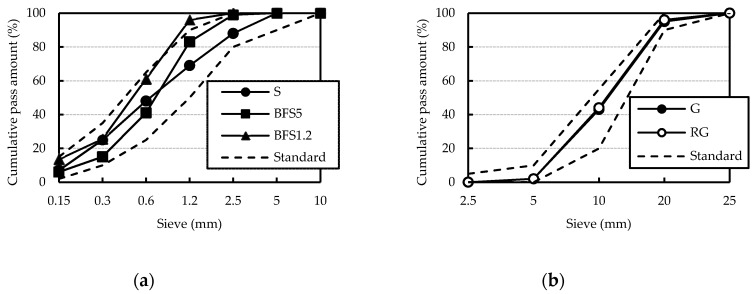
Particle size distribution: (**a**) Fine aggregate; (**b**) coarse aggregate.

The cement used was the JIS R 5210 standard Portland cement (OPC) and JIS R 5211 type B blast-furnace slag cement (BB). BB was blended cement with cement replacement levels of 30%–60% ground granulated blast furnace slag for mitigation of alkali–silica reaction. Blast furnace slag sand (BFS5, BFS1.2) was used. These were from Okayama Prefecture (Fukuyama City and Kurashiki City), Japan. These two slags have different particle sizes. Figure 1 shows BFS5 and BFS1.2. Recycled coarse aggregate (RG) was obtained from a prestressed concrete pile which had been crushed in a jaw crusher. Details of original concrete mix was unclear and further treatment was not carried out. After obtaining the RG, it was separated into different particle sizes measuring 5–13 and 13–20 mm, respectively. Then, it was blended at a ratio of 8:2 against RG volume. Figure 2 shows the RG. Crushed stone (G) was also blended at the same ratio as RG. SP and AEA were also added to improve workability and resistance to freezing and thawing. These materials and physical properties are presented in Table 1. Chemical compositions of cement and blast furnace slag sand are presented in Table 2 and Table 3. Additionally, the particle distribution curve of blast furnace slag sand and recycled coarse aggregate are presented in Figure 3.

### 2.2. Concrete Mix Design

All specimens and mix proportions are shown in Table 4. These mix proportions have constant unit weight of water and cement, and W/C was set as 47%, constantly. Replacement ratio of blast furnace slag sand was 30%, 50%, and 100%, respectively. The concrete was mixed by a twin shaft mixer for 3 min in a laboratory. During the mixing of BFS5-100R with OPC segregation was observed, and the slump did not satisfy the required valued (12.0 ± 1.0 cm). For this reason, the mix was excluded from tests described later. Target air content of fresh concrete was 6.0% ± 1.0%. The mixtures using RG were described by the symbol with the suffix “R”.

### 2.3. Tests

Concrete bleeding was tested according to JIS A 1123.

After 24 h from casting concrete, all specimens were demolded and cured in water at 20 °C ± 2 °C. The curing period for each test varied as described below.

Their compressive strength was tested according to JIS A 1108. Cylindrical specimens measuring 100 mm in diameter and 200 mm on height were used. Compressive strength tests of specimens were performed at 7 and 28 days, respectively.

**Figure 4:**
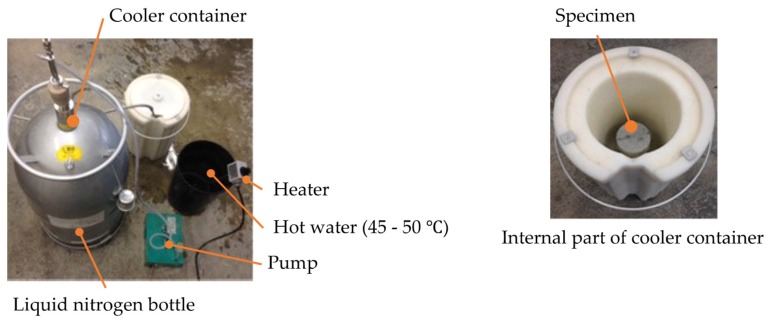
Equipment used for rapid freezing and thawing test using liquid nitrogen.

A rapid freezing and thawing test using liquid nitrogen was performed using equipment shown in Figure 4 [20,21,22,23]. This test was proposed by our research laboratory. The test can be performed in only one day and is more rigorous than JIS A 1148 (A method). The durability factor after 10 freeze-thaw cycles of the rapid freezing and thawing test tends to be lower than that after 300 cycles measured according to JIS A 1148 (A method). The test procedure is presented below.

**Figure 5:**
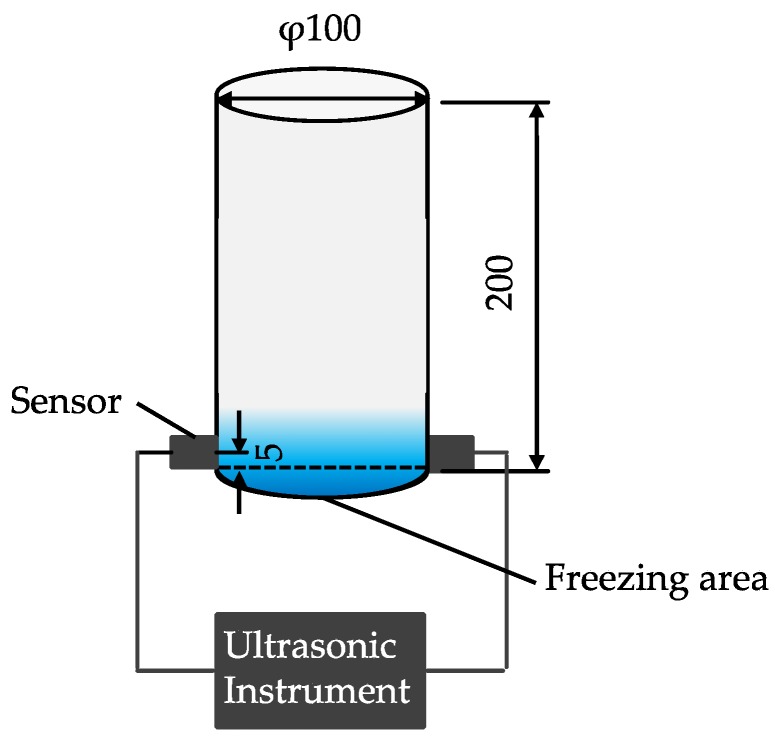
Outline of measuring for ultrasonic pulse time on specimen (mm).

**Figure 6:**
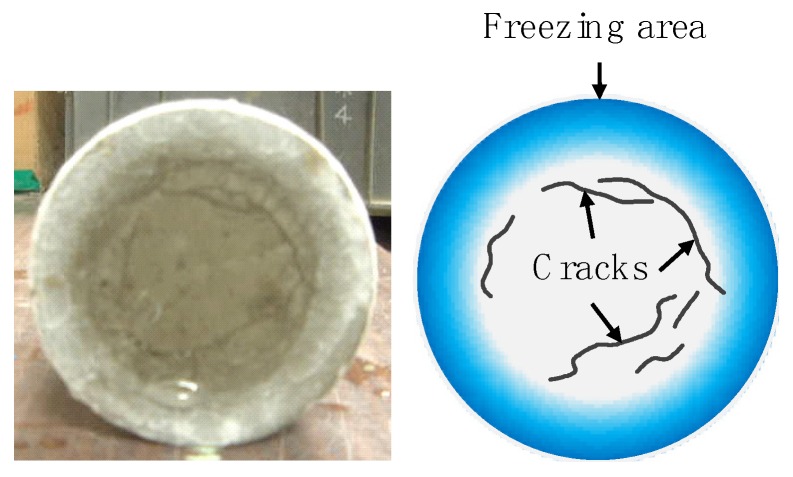
Circular cracks occurred on the bottom of cylindrical specimen by rapid freezing and thawing test.

A cylindrical specimen (100 mm diameter and 200 mm high) with an age of 28 days was placed in the center of a cooler container with a lid. Next, it was blown with liquid nitrogen for 30 s and then immersed in hot water with a temperature of 45 °C–50 °C for 5 min. After removing the specimen from hot water, a sensor was placed at a position of 5 mm height from the bottom of the specimen and ultrasonic pulse time was measured following ultrasonic testing as showed in Figure 5. The initial ultrasonic pulse time with a zero cycle was measured and then dynamic modulus of elasticity by ultrasonic pulse velocity was calculated. The situation of freezing and circular cracks that occurred on the bottom of cylindrical specimen is presented in Figure 6. This process was defined as one cycle, and until 60% or less, or until 10 cycles were reached. Equation (1) is used to calculate the dynamic modulus of elasticity of concrete.

(1) $$P_{n}=\frac{{\left(V_{n}\right)}^{2}}{{\left(V_{0}\right)}^{2}}\times 100$$

where, *P_n_*: Relative dynamic modulus of elasticity (%), *V*_0_: Ultrasonic pulse velocity at 0 cycle (km/sec.), *V_n_*: Ultrasonic pulse velocity after n cycle (km/sec.).

Here, mixes containing blast furnace slag cement type B showed poor performance in the rapid freezing and thawing test. Therefore, these mixes were tested according to JIS A 1148 (A method).

## 3. Results and Discussion

### 3.1. Bleeding

**Figure 7:**
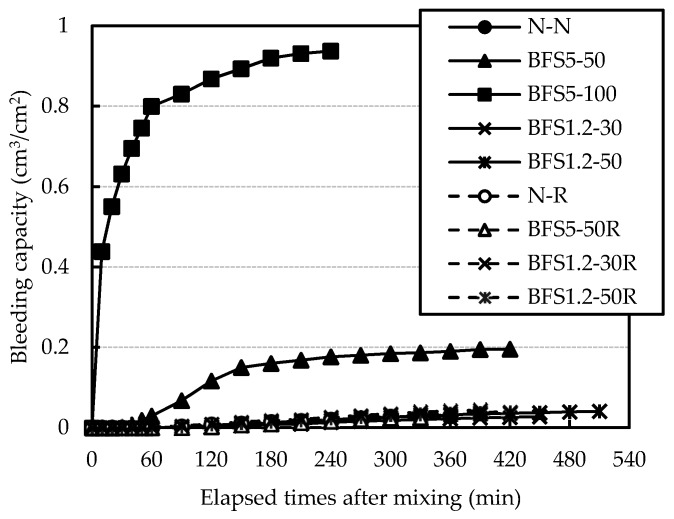
Relationship between bleeding capacity and elapsed times after mixing.

The relationship between bleeding capacity and elapsed times after mixing is shown in Figure 7.

**Figure 8:**
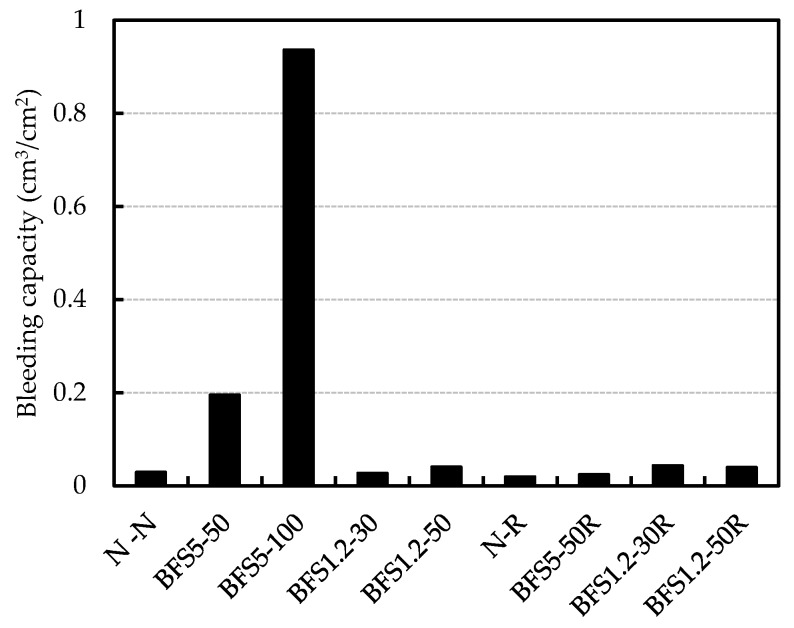
Final bleeding capacity.

The final bleeding capacity of concrete mixes with OPC is presented in Figure 8. In general, concrete mixes made with BFS tend to bleed more [24]. In this study, the result of BFS5-100 showed about 0.9 cm^3^/cm^2^ at 240 min. In particular, the bleeding capacity of BFS5-50 significantly exceeded the quality regulation of the bleeding capacity ≤0.3 cm^3^/cm^2^, which is limited by “Recommendation (draft) for shrinkage crack of reinforced concrete buildings” established by the Architectural Institute of Japan.

The final bleeding capacity for BFS1.2-50 was similar compared with N-N, the particle size of BSF1.2 was smaller than BFS5, and the viscosity of mortar became high. The bleeding capacities for mixes with RG were less than 0.1 cm^3^/cm^2^, regardless of type and blending ratio of BFS. It is assumed that bleeding water was retained by irregularities and fine particles coated on the surface of RG.

### 3.2. Compressive Strength

**Figure 9:**
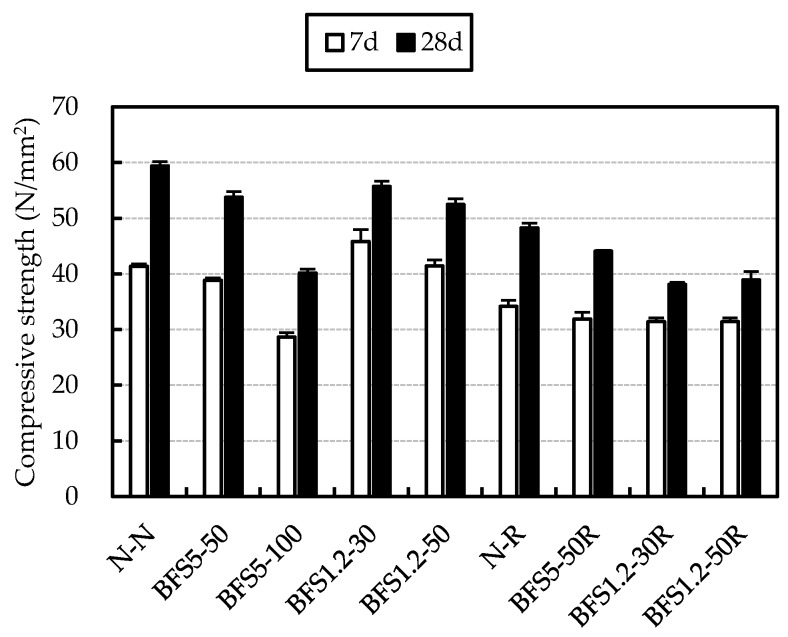
Compressive strength of concrete mixes with Ordinary Portland Cement (OPC).

The compressive strength for mixes with OPC is presented in Figure 9. It was evident that compressive strengths of specimens were reduced due to usage of RG and BFS. These reductions were 5–10 N/mm^2^ at seven days and 15–20 N/mm^2^ at 28 days.

When compared with N-R at seven days, strengths with RG and BFS were similar. At 28 days they decreased to 5–10 N/mm^2^. The reason for low strength of BFS5-100 was considered to be due to voids formation as a result of bleeding and decrease in interface adhesion between coarse aggregate and the mortar.

**Figure 10:**
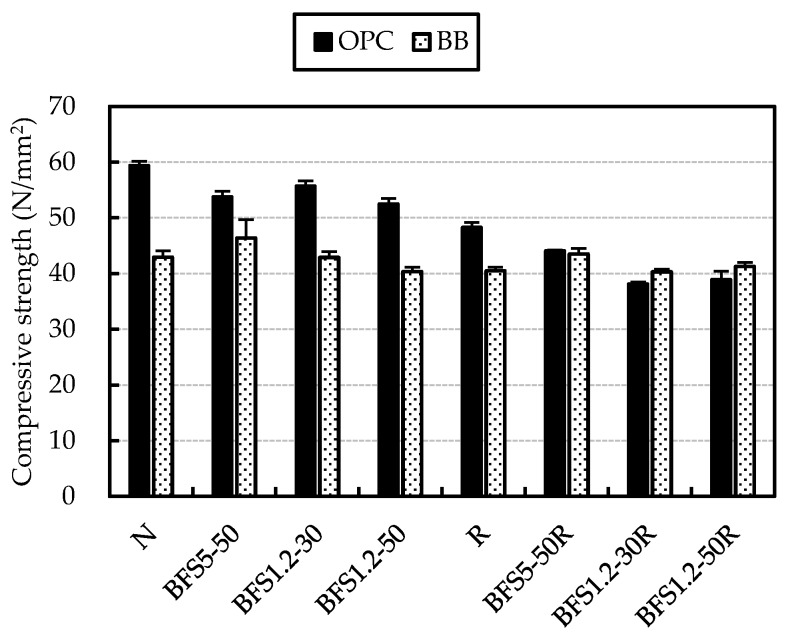
Compressive strength of concrete mixes with Ordinary Portland Cement (OPC) and Blast furnace slag cement type B (BB) at 28 days.

Figure 10 shows a comparison of strength for the OPC and BB-cement mixes at 28 days. BFS and G mixes with BB-cement had lower strengths compared with the OPC mixes. On the other hand, at 28 days, the strength of BFS and RG mixes with BB-cement were similar to mixes with OPC. This is thought to be due to the assumption that the interface between aggregate and cement paste was improved by latent hydraulicity between blast furnace slag and Ca(OH)_2_ remaining in the cement paste or coated mortar of RG.

### 3.3. Rapid Resistance to Freezing and Thawing

**Figure 11:**
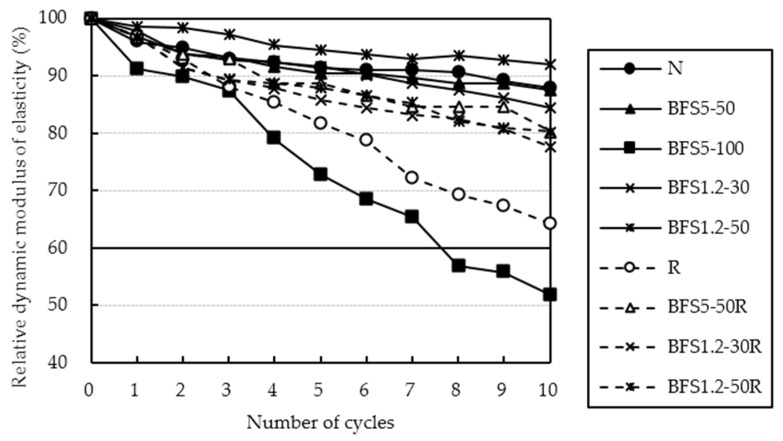
Relative dynamic modulus of elasticity after rapid freezing and thawing test for OPC mixes.

The relative dynamic modulus of elasticity obtained from the rapid freezing and thawing test for OPC specimens are presented in Figure 11. Relative dynamic modulus of elasticity of specimens with BFS showed a slight reduction without BFS5-100. Decreasing of entrained air in BFS5-100 was caused by bleeding. Therefore, freezing and thawing resistance of the specimen with BFS showed good results as the bleeding capacity was low.

**Figure 12:**
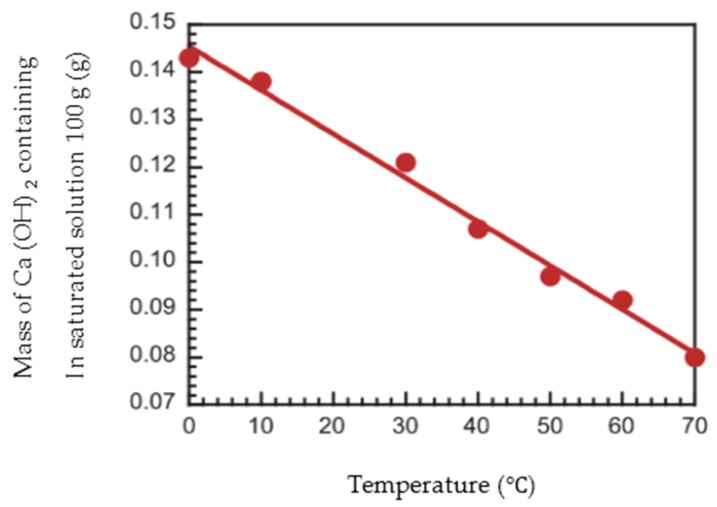
Temperature dependence of solubility of Ca (OH)_2_ [26].

Relative dynamic modulus of elasticity of RG and BFS mixes showed improvement, when compared to the concrete with RG only. For this reason, it is noted that Ca(OH)_2_ does not deposit around the aggregate [25] when BFS was used. This is shown in Figure 12. Solubility of Ca(OH)_2_ at 70 °C is about half of its value at 0 °C in Figure 11. The reason for this rather uncommon phenomenon is that the dissolution of Ca(OH)_2_ in water is an exothermic process, and it also adheres to Le Chatelier’s principle. A lowering of temperature thus favours the elimination of heat liberated through the process of dissolution and increases the equilibrium constant of dissolution of Ca(OH)_2_, and so increases its solubility at low temperature. This counter-intuitive temperature dependence of solubility is referred to as “retrograde” or “inverse” solubility. Ca(OH)_2_ deposits around the aggregate in ordinary concrete. Ca(OH)_2_ dissolves more easily in water at low temperature due to water being accumulated in a gap made by dissolved Ca(OH)_2_ [26]. When BFS was used, namely, Ca(OH)_2_ does not deposit around the aggregate. Moreover, it was shown that this is effective even for recycled concrete using low quality recycled coarse aggregates, which are not popular due to their assumed resistance to freezing and thawing.

**Figure 13:**
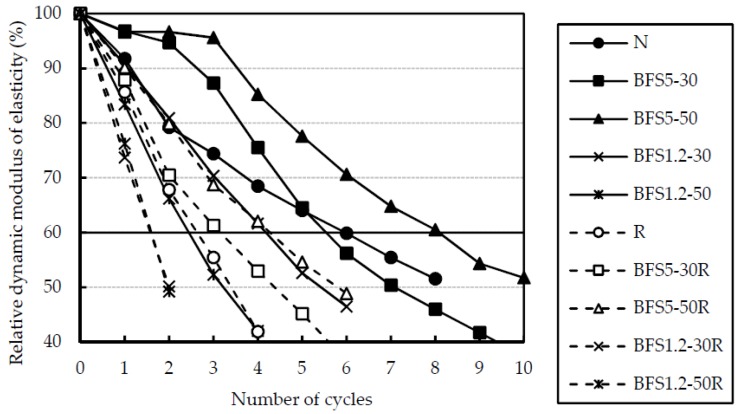
Relative dynamic modulus of elasticity after rapid freezing and thawing test for BB mixes.

**Figure 14:**
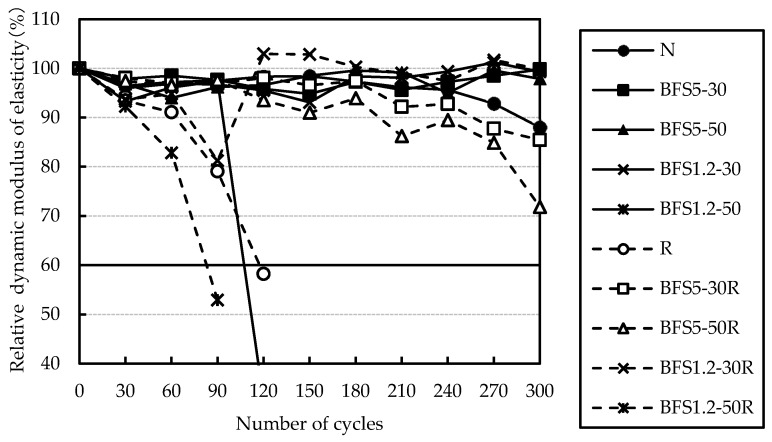
Relative dynamic modulus of elasticity after Japanese Industrial Standards (JIS) A 1148 (A method) for BB mixes.

Relative dynamic modulus of elasticity obtained from rapid freezing and thawing test for the BB-cement mixes are presented in Figure 13. Relative dynamic modulus of elasticity of all mixes with BB-cement and AEA decreased soon. The reason for this is not clear. The air void system was calculated using the method given in ASTM C 457 for loss of micro-air in AEA bleeding by mixing BFS that has a high density. Air void systems of a part mix with BB are shown in Table 5. The mixes had an air void system as shown by concrete with AEA. Therefore, all the mixes were examined by the JIS A 1148 A method. The relative dynamic modulus of elasticity obtained from the JIS A 1148 A method for the BB-cement mixes are presented in Figure 14. Freeze-thaw resistance for mixes with BFS tended to improve. However, with 50% BFS1.2, it decreased significantly regardless of coarse aggregate. It was assumed that freeze-thaw resistance improved due to latent hydraulicity of BFS with smaller particle size [27,28], but in this study, the effect did not appear.

## 4. Conclusions

The following conclusions were obtained from this study:

- Bleeding capacities were observed to be 0.1 cm^3^/cm^2^ or less when the mix ratio of blast furnace slag sand was 50% or less. It is thought that bleeding water was retained by irregularities and fine particles coated at the surface of RG.
- Compressive strength due to usage of RG and BFS was lower than that of the normal aggregate concrete. At 28 days the strength for the BFS and RG mixes with BB-cement were similar to mixes with OPC.
- Resistance to freezing and thawing was improved by mixing the blast furnace slag sand, and the relative dynamic modulus of elasticity of concrete using RG and BFS with OPC after the freezing and thawing test was approximately 80%.

Hence, it was found that mixing 50% blast furnace slag sand was effective for bleeding and resistance to freezing and thawing. In the future, we would like to further examine characteristics of strength and drying shrinkage. In addition to putting this into practical use for recycled aggregate concrete with blast furnace slag sand.

## Figures and Tables

**Table 1:** Material’s properties.

Type	Symbol	Physical Property
Cement	OPC	Ordinaly Portland cement (Density: 3.16 g/cm^3^, specific surface area: 3400 cm^2^/g)
	BB	Blast furnace slag cement type B (Density: 3.04 g/cm^3^, specific surface area: 3810 cm^2^/g)
Fine aggregate	S	Crushed sand (Density in saturated surface dry condition: 2.57 g/cm^3^, absorption: 1.77%)
	BFS5	Blast furnace slag sand made in Fukuyama (Density in saturated surface dry condition: 2.73 g/cm^3^, absorption: 0.30%)
	BFS1.2	Blast furnace slag sand made in Kurashiki (Density in saturated surface dry condition: 2.73 g/cm^3^, absorption: 0.40%)
Coarse aggregate	G	Crushed stone (Density in saturated surface dry condition: 2.57 g/cm^3^, absorption: 1.62%)
	RG	Recycled coarse aggregate (Density in saturated surface dry condition: 2.43 g/cm^3^, absorption: 6.20%)
Chemical admixture	SP	Polycarboxylate based superplasticizer
	AEA	Alkly based air entraining agent

**Table 2:** Chemical compositions of cement.

		Cement	
		OPC	BB
ig. loss	(%)	1.78	1.51
Insol.		0.17	0.21
SiO_2_		21.06	25.29
Al_2_O_3_		5.15	8.46
Fe_2_O_3_		2.80	1.92
CaO		64.17	55.81
MgO		1.46	3.02
SO_3_		2.02	2.04
Na_2_O		0.28	0.25
K_2_O		0.42	0.39
TiO_2_		0.26	0.43
P_2_O_5_		0.17	0.12
MnO		0.08	0.17
Cl		0.006	0.005

**Table 3:** Chemical compositions of blast furnace slag sand.

		Blast Furnace Slag Sand	
		BFS5	BFS1.2
CaO	(%)	41.8	43.9
S		0.80	0.65
SO_3_		0.02	0.03
FeO		0.50	0.28

**Table 4:** Mix proportions.

Symbol	Cement	W/C	Unit Content (kg/m^3^)							Slump	Air
	Type	(%)	W	C	S	BFS5	BFS1.2	G	RG	(cm)	(%)
N-N	OPC	47	165	350	802	-	-	905	-	13.0	4.9
BFS5-50					401	426				13.0	5.0
BFS5-100					-	852				11.0	5.0
BFS1.2-30					562	-	256			11.0	6.0
BFS1.2-50					401		426			13.0	7.0
N-R					802		-	-	855	11.5	5.0
BFS5-50R					401	426				11.0	7.0
BFS5-100R					-	852				2.5	5.0
BFS1.2-30R					562	-	256			13.0	6.0
BFS1.2-50R					401		426			13.0	5.5
B-N	BB				791		-	899	-	13.0	6.4
BFS5-30					554	259				12.0	6.3
BFS5-50					396	431				12.0	6.6
BFS1.2-30					554	-	254			13.0	5.7
BFS1.2-50					396		424			13.0	6.1
B-R					791		-	-	868	11.0	5.2
BFS5-30R					554	259				11.0	6.6
BFS5-50R					396	431				13.0	6.7
BFS1.2-30R					554	-	254			11.5	6.1
BFS1.2-50R					396		424			12.0	6.6

**Table 5:** Void spacing factor and average air diameter for BB mixes.

Symbol	Void Spacing Factor (μm)	Average Air Diameter (μm)
B-N	221.1	82.7
BFS5-30	320.6	119.2
B-R	193.8	73.1
BFS5-30R	159.5	74.6
